# Authors' Reply: Dermatologists' Way of Informative Content on Dermatology and Cosmetology on Social Media

**DOI:** 10.1111/jocd.71190

**Published:** 2026-09-16

**Authors:** Simge Ünal Işık, Neslihan Demirel Öğüt

**Affiliations:** ^1^ Department of Dermatology and Venereology, Faculty of Medicine Uşak Training and Research Hospital, Uşak University Uşak Turkey

**Keywords:** cosmetology, dermatology, digital platforms, social media, WhatsApp

Dear Editor,

We appreciate the interest shown in our recently published study and thank the authors for their thoughtful comments and for highlighting important aspects of dermatologists' use of social media [1]. We agree that the growing influence of digital platforms on health‐related behaviors requires a nuanced and multifaceted evaluation.

As noted, the use of self‐reported data represents a recognized limitation. Nevertheless, survey‐based studies remain a well‐established approach for exploring physicians' attitudes, motivations, and professional behaviors, particularly in the context of digital health communication. In a study conducted among dermatologists in Australia—based on the identification and evaluation of dermatologists' social media accounts and their posted content—social media use was shown to vary according to purpose, with LinkedIn and ResearchGate being used primarily for individual academic visibility, and Facebook and Instagram more commonly used at an institutional level for patient engagement [2]. These findings suggest that platform preferences may be shaped by the intended use. In contrast, our study did not identify such a distinction, and Instagram emerged as the most frequently used platform. This difference may reflect contextual variations in social media practices, as well as the fact that our study focused on dermatologists' self‐reported perspectives rather than direct analysis of online content.

As suggested by the authors [1], content analysis represents a valuable complementary approach. However, such analyses primarily address the characteristics of published content, whereas our study was designed to examine physicians' behaviors and awareness. For this reason, these approaches should be viewed as complementary rather than interchangeable.

The impact of social media on patient perception and behavior is also an important consideration. There is increasing evidence that online health information can significantly influence decision‐making processes and contribute to the spread of misinformation [3]. Moreover, younger individuals and those seeking cosmetic interventions have been reported to rely more heavily on online information sources [2]. In this context, integrating both physician and patient perspectives is essential. Our findings further underscore the need for dermatologists to maintain an active and visible presence on social media.

Ethical considerations—including patient privacy, informed consent, and transparency in content sharing—remain key challenges in this area. In our study, awareness of patient confidentiality and data protection regulations among dermatologists was found to be variable, suggesting that awareness of ethical and legal responsibilities related to social media use remains inconsistent. Previous studies have similarly highlighted concerns regarding professional boundaries, patient privacy, data protection, and commercially driven or potentially biased health information in social media–based health communication [4]. These findings underscore the need for clearer ethical guidance and increased awareness in this evolving field.

Finally, although our study was conducted among dermatologists in Turkey, regional, cultural, and legal differences in social media use underscore the importance of country‐specific data. Such contributions provide valuable context to the existing literature and support future comparative research. The points raised by the authors highlight the complex and multidimensional nature of social media use in dermatology. Our study contributes to this field by offering insights into dermatologists' perspectives and practices, while also emphasizing the need for future research integrating content analysis, patient‐centered outcomes, and ethical considerations to further advance understanding in this rapidly evolving area.

## Funding

The authors have nothing to report.

## Conflicts of Interest

The authors declare no conflicts of interest.

## Data Availability

Research data are not shared.
